# Editorial for the Special Issue on Flexible and Wearable Sensors

**DOI:** 10.3390/mi14071400

**Published:** 2023-07-09

**Authors:** Libo Gao, Zhuoqing Yang

**Affiliations:** 1 Pen-Tung Sah Institute of Micro-Nano Science and Technology, Xiamen University, Xiamen 361102, China; 2 Innovation Laboratory for Sciences and Technologies of Energy Materials of Fujian Province (IKKEM), Xiamen 361005, China; 3 National Key Laboratory of Micro/Nano Fabrication Technology, School of Electronic Information and Electrical Engineering, Shanghai Jiao Tong University, Shanghai 200240, China; yzhuoqing@sjtu.edu.cn

Flexible wearable sensors have garnered significant interest in the fields of human-computer interaction, materials science, and biomedicine. The remarkable progress achieved in combining flexible materials and sensor manufacturing processes has led to substantial growth in flexible wearable sensors, making it a highly attractive and rapidly evolving area of interdisciplinary research. This Special Issue aims to highlight the latest advancements, ongoing challenges, and emerging opportunities in flexible wearable sensors, encompassing both fundamental principles and practical applications. The objective of this Special Issue is to inspire the community by addressing key questions in this field, with the ultimate aim of maximizing the societal impact of flexible wearable sensors.

In this Special Issue on Flexible and Wearable Sensors, we have included 25 papers, including 24 research papers, covering applications in human-computer interaction [1,2,3], mechanical design [4,5,6,7,8], health monitoring [9,10,11,12,13,14,15], manufacturing technology [16,17,18,19,20], algorithms [21,22,23], and smart cities [24]. Additionally, we feature an intriguing review paper focusing on flexible wearable sensor devices for biomedical applications [25]. 

In particular, Xue et al. proposed a frictional electromagnetic hybrid harvester with a low starting wind speed and an engineering-practical propeller design approach to achieve output power over a wide range of wind speeds [24]. Vanhala et al. proposed a strategy for long carbon stitched fibers in the form of permeable carbon fiber cloth placed on a stretchable thermoplastic polyurethane matrix to improve the 3D printed matrix’s mechanical, electrical, and thermal properties [17]. Gao et al. proposed a flexible skin pressure sensor for monitoring the pulse of the radial artery, which was connected to a flexible processing circuit mount to enable real-time wireless, accurate monitoring of the pulse by a smartphone [12]. Zhang et al. combined 2D transition metal carbides, nitrides, and carbon-nitrides with a honeycomb structure formed by femtosecond filamentary pulses to design and fabricate a high-performance flexible piezoresistive sensor that enables stress-strain detection during human movement [14].

Finally, the review articles in this issue highlight the latest advances in flexible wearable sensor devices for biomedical applications [25]. As the field of flexible wearable electronics continues to grow, the number of relevant articles published on flexible wearable sensors is also increasing, and we look forward to the day when flexible wearable electronics move from the laboratory to full social life.

It is our hope that this Special Issue on Flexible and Wearable Sensors provides readers with valuable insights into the current state of the art in this rapidly evolving research area, presenting some of the latest technologies developed in the field.

## Data Availability

The data presented in this study are available on request from the corresponding author.

## References

[1] Nikitina N.A., Ryabkin D.I., Suchkova V.V., Kuksin A.V., Pyankov E.S., Ichkitidze L.P., Maksimkin A.V., Kitsyuk E.P., Gerasimenko E.A., Telyshev D.V. (2023). Laser-Formed Sensors with Electrically Conductive MWCNT Networks for Gesture Recognition Applications. Micromachines.

[2] Yu Q., Zhang J. (2023). Flexible Capacitive Pressure Sensor Based on a Double-Sided Microstructure Porous Dielectric Layer. Micromachines.

[3] Sha B., Lü X., Jiang L. (2022). High Sensitivity and Wide Range Biomimetic Tactile-Pressure Sensor Based on 2D Graphene Film and 3D Graphene Foam. Micromachines.

[4] Hubmann M., Bakr M., Groten J., Pletz M., Vanfleteren J., Bossuyt F., Madadnia B., Stadlober B. (2023). Parameter Study on Force Curves of Assembled Electronic Components on Foils during Injection Overmolding Using Simulation. Micromachines.

[5] Li B., Sun H., Zhang H., Li Y., Zang J., Cao X., Zhu X., Zhao X., Zhang Z. (2022). Refractive Index Sensor Based on the Fano Resonance in Metal&ndash;Insulator&ndash;Metal Waveguides Coupled with a Whistle-Shaped Cavity. Micromachines.

[6] Liao Z., Jing J., Gao R., Guo Y., Yao B., Zhang H., Zhao Z., Zhang W., Wang Y., Zhang Z. (2022). A Direct-Reading MEMS Conductivity Sensor with a Parallel-Symmetric Four-Electrode Configuration. Micromachines.

[7] Gao R., Zhang W., Jing J., Liao Z., Zhao Z., Yao B., Zhang H., Guo Y., Xu Y., Wang Y. (2022). Design, Fabrication, and Dynamic Environmental Test of a Piezoresistive Pressure Sensor. Micromachines.

[8] Liu M., Hu J., Zeng Q., Jian Z., Nie L. (2022). Sound Source Localization Based on Multi-Channel Cross-Correlation Weighted Beamforming. Micromachines.

[9] Zhang Z., Zhang H., Zhang Q., Zhao X., Li B., Zang J., Zhao X., Zhang T. (2023). A Pressure and Temperature Dual-Parameter Sensor Based on a Composite Material for Electronic Wearable Devices. Micromachines.

[10] Brendgen R., Graßmann C., Gellner S., Schwarz-Pfeiffer A. (2022). Textile One-Component Organic Electrochemical Sensor for Near-Body Applications. Micromachines.

[11] Miao X., Gao X., Su K., Li Y., Yang Z. (2022). A Flexible Thermocouple Film Sensor for Respiratory Monitoring. Micromachines.

[12] Zheng W., Xu H., Wang M., Duan Q., Yuan Y., Wang W., Gao L. (2022). On-Skin Flexible Pressure Sensor with High Sensitivity for Portable Pulse Monitoring. Micromachines.

[13] Shi S., Liang J., Qu C., Chen S., Sheng B. (2022). Ramie Fabric Treated with Carboxymethylcellulose and Laser Engraved for Strain and Humidity Sensing. Micromachines.

[14] Su Y., Ma K., Yuan F., Tang J., Liu M., Zhang X. (2022). High-Performance Flexible Piezoresistive Sensor Based on Ti_3_C_2_Tx MXene with a Honeycomb-like Structure for Human Activity Monitoring. Micromachines.

[15] Xu D., Duan L., Yan S., Wang Y., Cao K., Wang W., Xu H., Wang Y., Hu L., Gao L. (2022). Monolayer MoS_2_-Based Flexible and Highly Sensitive Pressure Sensor with Wide Sensing Range. Micromachines.

[16] Bakr M., Hubmann M., Bossuyt F., Vanfleteren J. (2022). A Study on Over-Molded Copper-Based Flexible Electronic Circuits. Micromachines.

[17] Salo T., Di Vito D., Halme A., Vanhala J. (2022). Electromechanical Properties of 3D-Printed Stretchable Carbon Fiber Composites. Micromachines.

[18] Pan X., Lin F., Wu C., Zeng Y., Chen G., Chen Q., Sun D., Hai Z. (2022). Additive-Manufactured Platinum Thin-Film Strain Gauges for Structural Microstrain Testing at Elevated Temperatures. Micromachines.

[19] Lin F., Pan X., Wu C., Zeng Y., Chen G., Chen Q., Sun D., Hai Z. (2022). ZrB2/SiCN Thin-Film Strain Gauges for In-Situ Strain Detection of Hot Components. Micromachines.

[20] Wu C., Lin F., Pan X., Zeng Y., Chen G., Xu L., He Y., Sun D., Hai Z. (2022). A SiCN Thin Film Thermistor Based on DVB Modified Polymer-Derived Ceramics. Micromachines.

[21] Zhu X., Ma Y., Guo D., Men J., Xue C., Cao X., Zhang Z. (2023). A Framework to Predict Gastric Cancer Based on Tongue Features and Deep Learning. Micromachines.

[22] Garzón-Posada A.O., Paredes-Madrid L., Peña A., Fontalvo V.M., Palacio C. (2022). Enhancing Part-to-Part Repeatability of Force-Sensing Resistors Using a Lean Six Sigma Approach. Micromachines.

[23] Li J., Zhang Z., Zhu X., Zhao Y., Ma Y., Zang J., Li B., Cao X., Xue C. (2022). Automatic Classification Framework of Tongue Feature Based on Convolutional Neural Networks. Micromachines.

[24] Li G., Cui J., Liu T., Zheng Y., Hao C., Hao X., Xue C. (2023). Triboelectric-Electromagnetic Hybrid Wind-Energy Harvester with a Low Startup Wind Speed in Urban Self-Powered Sensing. Micromachines.

[25] Nan X., Wang X., Kang T., Zhang J., Dong L., Dong J., Xia P., Wei D. (2022). Review of Flexible Wearable Sensor Devices for Biomedical Application. Micromachines.

